# Concerns Over Vuse e-Cigarette Digital Marketing and Implications for Public Health Regulation: Content Analysis

**DOI:** 10.2196/54661

**Published:** 2024-12-27

**Authors:** Eileen Han, Lauren K Lempert, Francesca Vescia, Bonnie Halpern-Felsher

**Affiliations:** 1 Center for Tobacco Control Research and Education, University of California, San Francisco, San Francisco, CA, United States; 2 REACH Lab, Department of Pediatrics, Division of Adolescent Medicine, Stanford University, Palo Alto, CA, United States

**Keywords:** e-cigarette, social media marketing, Vuse, adolescents and young adults, Food and Drug Administration, FDA, smoker, smoking, smoking device, tobacco, social media, Instagram, Facebook, promote, marketing, mobile phone

## Abstract

**Background:**

Electronic cigarettes (e-cigarettes) are the most used form of tobacco products among adolescents and young adults, and Vuse is one of the most popular brands of e-cigarettes among US adolescents. In October 2021, Vuse Solo became the first e-cigarette brand to receive marketing granted orders (MGOs) from the US Food and Drug Administration (FDA), authorizing its marketing and their tobacco-flavored pods. Vuse Ciro and Vuse Vibe, and their tobacco-only (“original”) e-liquids, were authorized for marketing in May 2022 and Vuse Alto tobacco-flavored devices were authorized in July 2024. These marketing authorizations are contingent upon the company adhering to the MGOs’ stated marketing restrictions, including reducing exposure and appeal to youth via digital, radio, television, print, and point-of-sale advertising.

**Objective:**

In this study, we analyzed the official social media channels of Vuse (Instagram and Facebook) to examine how Vuse marketed its products on social media and whether these marketing posts contain potentially youth-appealing themes.

**Methods:**

We conducted content analysis of the official RJ Reynolds Vapor Company Instagram and Facebook accounts. We collected all posts from October 10, 2019, when RJ Reynolds Vapor Company submitted its premarket tobacco product application to the FDA, to February 21, 2022, to cover the first winter holiday season after the MGO. Two coders developed the codebook with 17 themes based on the Content Appealing to Youth index to capture the posts’ characteristics and potentially youth-appealing content. We calculated the percentage of posts in which each code was present.

**Results:**

A total of 439 unique posts were identified. During this study’s period, there were no posts on Instagram or Facebook marketing Vuse Solo (the authorized product at that time). Instead, Vuse Alto (unauthorized to date of study) was heavily marketed, with 59.5% (n=261) of the posts specifically mentioning the product name. Further, “Vuse” more generally was marketed on social media without differentiating between the authorized and unauthorized products (n=182, 41.5%). The marketing messages contained several potentially youth-appealing themes including creativity or innovation (n=189, 43.1%), individuality or freedom (n=106, 24.2%), and themes related to art (n=150, 34.2%), music (n=77, 17.5%), sports (n=125, 28.5%), nature (with n=49, 11.2% of the posts containing flora imageries), alcohol imagery (n=10, 2.3%), and technology (n=6, 1.4%).

**Conclusions:**

Although Vuse Alto e-cigarettes had not yet obtained FDA marketing authorization during the 28 months of data collection, they were the primary Vuse e-cigarette devices marketed on social media. Vuse social media posts use themes that are appealing to and likely promote youth use, including creativity and innovation, individuality or freedom, arts and music, nature, technology, and alcohol imagery. The FDA should (1) prohibit companies from comarketing unauthorized products alongside authorized products, and (2) exercise enforcement against even authorized products that are marketed using youth-appealing features.

## Introduction

Electronic cigarettes (e-cigarettes) remain the most used tobacco product among adolescents, with 2 million middle- and high-school students reporting using e-cigarettes in 2023 [1]. Different brands of e-cigarettes over the last 10 years have had more or less popularity, starting with JUUL’s surge in 2017 when 10% of adolescents reported using JUUL [2] and they had over 70% of the e-cigarette market share in late 2018 through early 2019 [3,4].

Vuse-branded e-cigarettes were launched in 2013 by the British American Tobacco, owned by RJ Reynolds Vapor Company (RJRV). The Vuse family of products, including Vuse Solo, Alto, Ciro, and Vibe, has gained in popularity, with data from the National Youth Tobacco Survey showing that Vuse are some of the most commonly used e-cigarettes among adolescents, with 20.7% of adolescents having reported using Vuse in 2023 [1]. According to a CDC report based on retail data, Vuse ranked among the five top-selling brands during the 4-week period ending in December 2022 [5]. Vuse market share reached 42.1% as of November 2023, according to Nielsen convenience store reporting [6].

Vuse Solo was also the first e-cigarette to be authorized by the US Food and Drug Administration (FDA) through the premarket tobacco product application (PMTA) process [7,8], with the FDA issuing this first marketing granted order (MGO) for Vuse Solo in October, 2021. The law requires companies to first obtain marketing authorization from the FDA based on a previously submitted PMTA before coming to market [9,10]. For e-cigarette products that were on the market as of August 2016, companies were required to submit PMTAs by September 2020 to legally remain on the market. The FDA announced it would prioritize enforcement against e-cigarettes marketed without FDA authorization: (1) that were flavored, cartridge-based products (other than tobacco- or menthol-flavored); (2) for which the company failed to prevent youth access; or (3) that targeted minors or whose marketing is likely to promote use by minors [11].

The FDA’s 2021 MGO for Vuse Solo specifically only authorized the sale of Vuse Solo “original” tobacco-flavored pods and did not authorize the sale of menthol-flavored pods or other flavors. Two other Vuse e-cigarettes, Vuse Ciro and Vuse Vibe, and tobacco-flavored (“original”) Vuse e-liquids were authorized in May 2022 [12]. In October 2023, the FDA issued marketing denial orders to RJRV for six of their flavored Vuse Alto products, including three menthol-flavored products [13]. RJRV subsequently obtained a court order staying enforcement of Vuse Alto Menthol Pods pending further review [14]. On July 18, 2024, the FDA announced that it authorized the marketing of seven tobacco-flavored Vuse Alto e-cigarette products [15]. Sales data indicated that before obtaining the MGOs, Vuse Alto represented about 95% of the total sales of Vuse and remained on the market, while Vuse Solo, the first authorized product, had almost no market share [16]. As of today, the FDA has not authorized any Vuse Alto or Vuse Solo menthol e-cigarette product [12].

The Vuse Solo MGO indicated that marketing authorization is contingent upon RJRV adhering to the FDA’s stated conditions and marketing restrictions including reducing exposure to youth via digital, radio, television, print, and point-of-sale advertising, and not targeting youth. This restriction is important given that for decades the tobacco industry has deliberately targeted marketing to adolescents and young adults to expand their sales and increase the number of addicted customers [17], and more recently e-cigarette companies have adopted both these traditional and newer social media marketing tactics that appeal to youth [18-20]. Marketing strategies traditionally used by tobacco companies to target youth include using cartoons [21]; flavors such as candy and desserts, themes of freedom, bright colors, sleek design, technology-like appearances, young-looking models [22]; and music, arts, and design [23,24]. Indeed, one of the most popular e-cigarette brands of all time, Juul, used specific colors, music, and younger models in their advertisements to appeal to youth [25]. Social media marketing has become a widely used approach to marketing e-cigarettes, with studies clearly showing that marketing of e-cigarettes on social media influences youth use [18,26,27].

**Figure 1.**
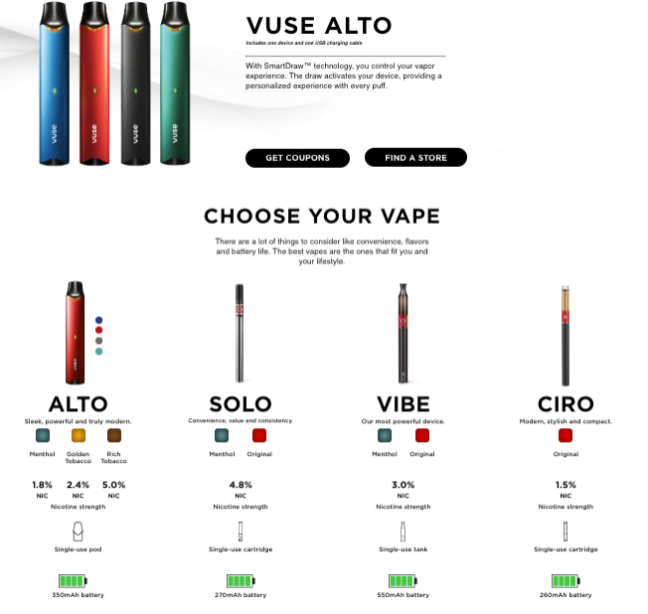
Image from the Vuse Vapor website captured on November 9, 2023, showing that Vuse Alto, which was not authorized until July 2024, was comarketed with authorized Vuse products (Solo, Vibe, and Ciro Original) [28].

It is important to determine whether RJRV’s marketing of Vuse on social media targets youth or includes youth-appealing themes despite the MGO conditions. Such information should inform the FDA’s post-MGO surveillance and actions, especially given that the FDA has the authority to withdraw marketing authorization if a company fails to meet the conditions stated in the MGOs and is required to withdraw the order if the continued marketing of the product “no longer is appropriate for the protection of the public health” [APPH] [10]. Further, Vuse is one of the few e-cigarette brands to continue marketing their products using an official account on social media. Moreover, RJRV has been comarketing several different Vuse products on their website, including those that have been authorized (eg, Vuse Solo “original”) and those that have not been authorized (eg, Vuse Alto and Vuse Alto Menthol, Figure 1). For example, Figure 1 shows the Vuse website after Vuse Solo was authorized by the FDA but before Vuse Alto was authorized, showing comarketing of authorized and unauthorized products.

We conducted a content analysis of Instagram and Facebook posts for all Vuse products over a 28-month period (October 2019 to February 2022), including both after the Vuse PMTA was submitted and before and after Vuse Solo was authorized, to address the following two questions: (1) what are the specific Vuse brands being marketed on official social media channels over this time period? and (2) do posts about Vuse products on Facebook and Instagram contain potentially youth-appealing themes? Findings from this study should help inform the FDA’s determination of whether the marketing of Vuse continues to be appropriate for public health (the regulatory standard) and inform the FDA’s future marketing decisions for other e-cigarette products.

## Methods

### Study Design

We collected and analyzed all posts on Instagram (@vusevaporus) and Facebook (Vuse Vapor US), from October 10, 2019, when RJRV submitted its PMTA application to the FDA, to February 21, 2022, about 4 months after the FDA issued the MGO for Vuse Solo (October 12, 2021) allowing us to collect data spanning over winter holidays as that is when companies often do a larger marketing push.

Instagram and Facebook were chosen as the social media platforms from which to collect the data because these were two major platforms where RJRV, the manufacturer who obtained marketing authorization for Vuse products, has actively posted marketing content and their official accounts can be easily identified and distinguished from posts by the general public.

We took screenshots of every post we collected in case some posts were later deleted or edited. A total of 627 posts were collected and coded. As the company often cross-posts contents on Facebook and Instagram, we included 439 unique posts for our final analysis.

### Coding

Our codebook was adapted from previous studies drawing on the Content Appealing to Youth (CAY) index on youth-appealing features of alcohol and e-cigarette advertisements [20,22,29]. The CAY index contains a wide variety of content characteristics that were specifically designed to attract young audiences. It has a few large categories that incorporate production techniques (a variety of audio-visual effects of the advertising media), character appeal (such as the presence of younger models, animated characters, and influencers), youth-oriented genres (such as humor, magic, or fantasy), situations and activities that are particularly attractive to youth (such as parties, social gathering, and concerts), product features (such as bright colors, sleek designs, flavors, and technology-like appearance), and rewarding appeals (products associated with being successful, rebellious, or gaining status) [30]. Our codebook drew on the existing codebook to identify overall characteristics of the Vuse products, in which youth-appealing features informed by literature may be found [29]. We developed the codebook iteratively as the two coders coded together, while deliberating on the features to be captured and the codes to use. We identified the race and ethnicity of people who were present in the posts through investigator observation.

A total of 159 codes were developed to analyze the posts, which were then combined into 17 themes. The codes represent characteristics of each post, with each post potentially having multiple codes. The following categories and individual codes in our codebook were expected to have potentially youth-appealing features: (1) flavors [31,32], (2) presence of human (perceived younger [22]), (3) promotional incentives (price reduction [33,34] or sweepstakes [35]), (4) product or use cues (having placed together with technological devices or customized design of packaging or wraps [22]), (5) activities (sports, dancing, partying, art, or music [23,24]), (6) setting (great outdoors [35]), (7) imageries (flora or fruit [35,36]), (8) environmental and social justice [35], and (9) rewarding appeals (positive sensation and mood promotion [37], and individuality or freedom [29]).

The full list of themes, definitions, codes, and examples can be found in Table 1. Codes that were expected to have potentially youth-appealing features are noted in italics.

**Table 1.** List of themes that were coded from the collected and analyzed posts, as well as definitions and examples of each theme.

Themes (N=17)	Definitions and codes associated with each theme	Examples of each theme (texts in caption or description of the image)
Vuse product family mentioned or shown	The mention of the exact name of one or more Vuse products (Vuse Alto, Vuse Solo, or others) or the presence of a device known as one of the Vuse products.	A post with a Vuse Alto device in it.
Colors	Any time “color,” “colors,” or a specific color name appears.	“Reimagine fancy with a bit of flair. Get the Alto in rose gold today. #VuseUSChargeBeyond”
Flavors	Any mention of flavors, whether it is general or a specific flavor.	“Feel the iconic flavor of Menthol. Order yours at the bio link…”“Your favorite flavor pods are now just $9.99. Order online and explore your passions right from your own element. Link in bio to buy.”
Humans	Post contains people with full or partial body image (real or drawn). Perceived gender (male, female, both, or unclear, present=1, not present=0), race (White=1, non-White=2, White and non-White=3), and *perceived age of the human* (*younger than 25 years*=1, older than 25 years=2, since Instagram has an age restriction for accessing contents from tobacco companies) were also coded.	A post showing someone’s hand holding a device.
Animals	The presence of actual animals or artwork of animal figures in the setting or on the device.	A wrap of the device with bird pattern on it.
Policy mentioned	Any mention of an existing, forthcoming, or potential policies related to e-cigarette^a^ products.	“Protect your right! Vote ‘NO’ on the proposed vapor menthol ban. Make your voice heard now by voting here: [link].”
Pandemic reference	Any post explicitly or implicitly referencing the COVID-19 pandemic, such as referring to e-cigarette use as a way to pass the time while stuck at home, mentioning attending events virtually that would typically be in person, or being able to travel again.	“Go wherever, see whatever and enjoy your Alto somewhere different. Take 20% Off Sitewide for your reunion with the world! Use code REUNITED. http://ms.spr.ly/6187V4dJV #VuseUSChargeBeyond”
Promotional incentives	Incentives for consumers to use the product or engage with the brand, coded as: sweepstakes, giveaways, *price reduction*, holiday sale, subscription, *cosponsored events*, and free delivery [36].	“VUSE Alto is available online - Not a subscriber? Get 10% off your first order! Use code EXTRA10 at checkout. Conditions apply. See website for full offer details.HAPPY BLACK FRIDAY. Go online now to get 30% off storewide and free shipping. Conditions apply. See website for full offer details.”
Product or use cues	Information about the product that could trigger cravings or facilitate actual use, coded as: product availability, price shown, packaging shown, *wraps pictured (customized design)*, Vuse shown with alcohol or with *technological devices*, and so on [36].	“Gold alto ✓ Golden Tobacco flavor pod ✓ Golden whiskey ✓ What’s your favorite happy hour combination? Let us know below 👇 #VuseUSChargeBeyond”
Adertisement-level descriptors	Listed terms (exact text or variations) that appeared in the post, depicting the characteristics of the products excluding flavors. Examples are: fruity, juicy, green, bold, deep, fresh, and sweet [36].	“Be a voice, not an echo. Fresh design. Same smooth, quiet draw. Link in bio to shop.”
Claims	Post has message about certain qualities that the Vuse product has, such as reduced harm, rechargeable, high quality, unique, new, etc [29]	“The Alto squeezes a 350mAh battery inside its smooth metal casing to power your inspiration all day long. #VuseUSChargeBeyond”
Activities	Product is associated with specific activities, including featuring activity-associated paraphernalia, or characters engaging in the activity. Examples are *art, sports or athletics*, manual labor, *music, partying*, gambling, drinking alcohol, etc [36]	“From blank canvas to original creation. See tattoo artist Zulu + chain stitch embroidery artist Taylor Rice collab. Dropping soon #VuseUSChargeBeyond”
Setting	Depiction of the primary location in which the main activity or an announced event happens [36]. It does not need to have people doing the activity but can be a setting that is typical or ideal for a kind of activity. We included artistic representations of settings, not just photographs or video. Examples are city, casino, farm, great outdoors, island or tropical, etc.	“LIVE MUSIC RETURNS 11/20. For our people & the love of live music, we’re rockin’ the NYC skyline via live-stream w/ @xxxxx. You ready? #vuserooftopsessionsus”
Imagery	Specific portrayals of images in the post [29] without also showing Vuse; the image has a symbolic meaning, codes included *flora*, flags, *fruit*, water, money, alcohol, motorcycle, and tobacco.	A post with image of trees and waterfall.
Warnings and disclaimers	Presence of disclaimers or warnings about age restriction to purchase, visit websites or social media, and addictiveness of nicotine.	Banner on top of the post warning about nicotine content and its addictiveness.
Environment and social justice	The claim that the company is committed to doing good to society, such as contributing to environmental protection or social justice.	“Vuse Alto is the first-ever carbon neutral vapor brand! See all the changes Vuse is making to become a more sustainable brand at the link in our bio.”
Reward appeals	Advertisement associating the product with attributes of positive life experiences. Examples are positive sensations or mood promotion, negative sensations or mood avoidance, physical performance, individuality or freedom, personal achievement and success, comraderies or friendship, social positioning, and appearance [23]. We added creativity or innovation.	“Sometimes going beyond doesn’t take going anywhere. Explore your passions, one personal space at a time. #VuseUSChargeBeyond”“We can see those creative wheels churning already. Share your own tips below 👇 #VuseUSChargeBeyond”

a e-cigarette: electronic cigarette.

Most of the codes were binary (0 as not present and 1 as present), with a few exceptions for nonbinary options (for example, for perceived race or ethnicity of humans in the advertisement, 1=White, 2=non-White, 3=both White and non-White), or when free text entry was appropriate.

We had two coders (EH and FV) for this study, with agreement on the codes by both coders. Once the 627 posts were collected, we randomly selected 25% (n=157) of the advertisements for coding by both coders, to finalize the codes and establish reliability.

We reached a mean percentage of agreement as 96.2%, after which we deliberated on our disagreements, and independently coded the remaining 75% (n=470) of the posts. The average Krippendorff α is .95) [38,39], with individual codes ranging from 0.67 to 1. We only included unique posts (N=439) for the final analysis.

### Data Analysis

After coding was complete, we calculated the percentage of the posts for each code.

### Ethical Considerations

This study does not involve human participants and instead is just a content analysis of existing social media marketing advertisements. This study is based on publicly available social media posts from official accounts of an e-cigarette company. Only the posts themselves were analyzed. The comments made by users were not analyzed, and when they were shown, were deidentified, so as not to reveal identities. Therefore, ethics board approval was not sought.

## Results

### Overview

Table 2 shows the percentage of the presence of each of our themes, which are further explained next.

**Table 2.** Percentages of each of the themes seen in the analyzed social media posts.

Themes		Quantity (N=439), n (%)
**Vuse product family mentioned or shown** ^**a**^		
General references to Vuse		182 (41.5)
Specified Vuse roduct (Vuse Alto)		261 (59.5)
Specified Vuse product (Vuse Vibe)^b^		1 (0.2)
Specified Vuse product (Vuse Solo)		0 (0)
Colors		40 (9.1)
**Flavors**		144 (32.8)
General flavor mentions		63 (14.4)
Other flavors		5 (1.1)
Menthol mentions		34 (7.7)
Presence of humans		64 (14.6)
Presence of animals		14 (3.2)
Policy mentioned		2 (0.5)
Pandemic reference		1 (3)
**Promotional incentives**		265 (60.4)
Events (cosponsoring)		99 (22.6)
Collaboration		57 (13)
Price reduction		32 (7.74)
Holiday sale		31 (7.1)
Sweepstake		31 (7.06)
Subscription		23 (5.2)
Free delivery		10 (2.3)
**Product or use cues**		
Power unit shown		183 (41.7)
Product availability		135 (30.8)
Customized wraps		85 (19.4)
Presence of pods		53 (12.1)
Packaging shown		41 (9.3)
Product in use		33 (7.5)
Price shown		15 (3.4)
With consumer products		14 (3.2)
With alcohol		10 (2.3)
Discrete or disguised use		8 (1.8)
With technological devices		6 (1.4)
**Advertisement-level descriptors**		95 (21.6)
New		64 (14.6)
Fresh		27 (6.2)
Rich		14 (3.2)
Smooth		9 (2.1)
Green		5 (1.1)
**Claims**		
Savings		25 (5.7)
Quality of e-liquids		11 (2.5)
Unique		7 (1.6)
**Activities**		
Art		150 (34.2)
Sports or athletics		125 (28.5)
Automobile		99 (22.6)
Music		77 (17.5)
Drinking alcohol		16 (3.6)
Relaxing		13 (3)
Vacationing		10 (2.3)
Partying		10 (2.3)
Manual labor		8 (1.8)
Being romantic		5 (1.1)
**Setting**		
Sporting event		73 (16.6)
City		46 (10.5)
Great outdoors		7 (1.6)
Island or tropical		5 (1.1)
**Imageries**		
Flora		49 (11.2)
Alcohol		10 (2.3)
Water		10 (2.3)
Flag		6 (1.4)
Presence of warnings and disclaimers		435 (99)
Environmental and social justice		14 (3.2)
**Rewarding appeals**		
Creativity or Innovation		189 (43.1)
Individuality or freedom		106 (24.2)
Positive mood promotion		28 (6.4)
Achievement or success		23 (5.2)
Adventure or spontaneity		17 (3.9)
Positive sensation promotion		13 (3)
Addiction (dependence)		11 (2.5)
Negative mood avoidance		6 (1.4)
Comraderies or friendship		5 (1.1)

a Product family was one code.

b The only post with Vuse Vibe was also featured with Vuse Alto.

### Product Family

We examined social media marketing posts for all products in the Vuse product family, including those that have been authorized (eg, Vuse Solo “original”) and those that have not obtained MGOs (eg, Vuse Alto Menthol). We found that 41.5% (182/439) of posts referred generally to “Vuse” without specifying a particular Vuse product (eg, Solo, Alto, Ciro, and Vibe), and 59.5% (261/439) specifically featured Vuse Alto. One post (0.2% of posts analyzed) featured Vuse Vibe together with Vuse Alto. There were no posts featuring Vuse Solo. Therefore, our findings focus on the marketing and posts for Vuse Alto if not otherwise specified, and on the Vuse Solo MGO when marketing authorization was mentioned.

### Colors

A total of 9.1% (40/439) of the posts mentioned colors of the device, either a theme color or a variety of colors for choice.

### Flavors

A total of 32.8% (n=144) of the 439 posts mentioned flavors, including 14.4% (n=63) mentioning flavors in general, 7.7% (n=34) mentioning menthol, and 5 mentions of other flavors (eg, “mixed berry,” 1.1%).

### Presence of Humans

A total of 14.6% (64/439) of the posts contained humans (either parts of the human body or full bodies). For those advertisements featuring humans, the majority had people who appeared to be older than 25 years.

### Promotional Incentives

A total of 60.4% (265/439) of the posts used promotions. The most common promotion method was cosponsoring an event, including car racing, concerts, and arts (99/439, 22.6%).

### Product or Use Cues

Of the 439 posts, a total of 41.7% (n=183) had the power units of the device presented. The power unit is the body of the device and can have different colors that were also highlighted in the posts. The presence of power units helped us identify the products being featured in the posts. A total of 19.4% (85) of the posts showed customized or specially designed wraps of the power unit. Other notable product or use cues included the presence of pods (n=53, 12.1%), product being in use (n=33, 7.5%), and product placed with other consumer products (eg, coffee: n=14, 3.2%), alcohol (n=10, 2.3%), discrete or disguised use (n=8, 1.8%), and technological devices (n=6, 1.4%).

### Advertisement-Level Descriptors

Of the 439 advertisements, 21.6% (n=95) used advertisement-level descriptors. Overall, only a small number of descriptors were used. The most frequently used descriptor was the product being “new” (n=64, 14.6%). Other than that, only a few descriptors were present, with relatively low percentage overall, such as being “fresh” (n=27, 6.2%) and “rich” (n=14, 3.2%). A few posts also featured the product being “green” (n=5, 1.1%).

### Claims

A total of 9.8% (n=43) of the 439 posts had claims. The most common claim was about savings (n=25, 5.7%) and the quality of e-liquids (n=11, 2.5%). Another noticeable claim was the product being “unique” (n=7, 1.6%). There were no indications of health-related effects or mentions of FDA authorization in any post.

### Activities

The most common activity depicted was art (150/439, 34.2%), sports or athletics (mostly car racing: 125/439, 28.5%), and music (77/439, 17.5%). Other noticeable activities featured included drinking alcohol (16/439, 3.6%) and vacationing (10/439, 2.3%).

### Setting

The most common setting of the 439 advertisements was sporting event (n=73, 16.6%), followed by city (n=46, 10.5%).

### Imageries

A total of 19.6% of the posts used identifiable imageries. Flora was the most commonly used imagery (n=49, 11.2%). Other noticeable imageries that were present included alcohol (n=10, 2.3%) and water (n=10, 2.3%).

### Presence of Warnings and Disclaimers

Most (435/439, 99%) of the advertisements had warnings and disclaimers about nicotine content and age restrictions for purchasing the product.

### Environmental and Social Justice

A total of 3.2% (14/439) of the posts featured environmental and social justice. We have seen posts talking about Vuse as a carbon-neutral product, its commitment to sustainability, and to helping veterans.

### Rewarding Appeals

The most common rewarding appeal depicted in the 439 posts was creativity or innovation (n=189, 43.1%), followed by individuality or freedom (n=106, 24.2%).

## Discussion

### Principal Results

Vuse Solo was the first e-cigarette product to obtain FDA marketing authorization, and “Vuse”-branded advertisements posted on social media provide a first glimpse of how social media marketing may be used for e-cigarette products that have received FDA MGOs. We found that while Vuse Solo received the FDA’s marketing authorization, it never appeared on the official Instagram and Facebook accounts, the only two active official accounts of RJRV, during the period of our study. Instead, Vuse Alto, which was not yet authorized by the FDA during the 28 months of data collection and analysis, was consistently identified in posts on both Facebook and Instagram accounts, and was the only product marketed on those platforms. Importantly, the products marketed on these two platforms were referred to generally as “Vuse,” rather than specifying “Solo” or “Alto.” The most commonly featured themes included creativity or innovation, art, sports or athletics, and individuality or freedom [20]. Vuse also sponsored car racing and art events frequently during the period of study. These contents have been used by tobacco companies for a long time [23,24,40,41], and according to CAY index, they are appealing to youth [20,30]. Other themes that are potentially appealing to youth, including the copresence of alcohol and technological devices [20,42,43], and the featuring of nature themes, were also found.

### Limitations

Our analysis was based on two platforms where the products have been marketed. The posts were collected from the official account of the company (RJRV) that sells these products, and did not include other unofficial channels that are possibly marketing these products with possible youth-appealing strategies that we did not identify in this study [44]. Instagram has age restrictions on contents posted by RJRV that limited the access of users aged younger than 25 years. While users are able to circumvent such restrictions, the likelihood of the contents reaching underaged users may be lower than those user-generated contents without age restrictions. The analysis only focused on the contents of the posts marketing the products but cannot assess users’ perceptions of the contents. Future research could examine more user-generated content related to Vuse products.

### Potentially Youth-Appealing Images and Themes, as Defined by the CAY Index

Our analysis of the Vuse social media marketing posts indicate a number of potentially youth appealing images and themes, as defined by the CAY index, the existing literature on youth-appealing advertisements [30,45,46], and as described by the FDA [47].

First, the most common social media marketing themes included art and music, featuring creativity and self-expression, all of which are considered youth-appealing by the CAY index [20]. Music [24], art, and design [23] have traditionally been used by major tobacco companies to promote cigarettes to young adults and “hipsters,” hoping that this marketing would spread the trend to a wider variety of younger users [23]. Similarly, RJRV featured sponsored events such as rooftop concerts and music festivals, activities that are youth oriented and likely to reach young consumers and influence use and are among the content that RJRV said in its PMTA that it would not use in its marketing to reduce youth appeal [7]. In addition to art and music, Vuse promotional posts featured themes of creativity and innovation and self-expression (customizing the devices with different designs of wraps, using the devices wherever one feels good), which are all recognized to be youth-appealing features in e-cigarette marketing [18,20].

Second, RJRV’s social media promotional activities for Vuse also included sponsorship of car racing games and featured artists and designers working to design race cars. The MGO said RJRV could mitigate risks to youth by not using marketing content that featured this kind of sports activity [7]. Tobacco companies have historically been major sponsors of car racing [48], and recent surveys showed that car racing events gained more viewership of younger audiences including adolescents [49,50]. In our findings, Vuse-sponsored car racing posts emphasize creativity through featuring collaboration with artists and designers and audience-engaging activities such as submitting their own design work to win prizes, which have the potential to attract creative-minded youth.

Third, while a smaller percentage, we observed posts featuring alcohol with Vuse, and placing technological products such as smartphones with Vuse devices. Couse of alcohol and tobacco products, including e-cigarettes, is associated with concerning health impacts [51,52], and the trend of concurrent or subsequent use of cigarettes or e-cigarettes and alcohol use among adolescents and young adults is disturbing [43,53]. The placement of Vuse with digital or technical products is a tactic frequently used by tobacco companies to target adolescents by promoting e-cigarette devices as cool technology with sleek designs and technology-like appearance [54-56]. Youth are attracted to e-cigarettes designed to be sleek in shape that can be used discreetly [57]. Indeed, JUUL was marketed as the “iPhone of e-cigarettes” and its meteoric rise in popularity was connected to this sleek design [42].

Our findings also showed the presence of nature-related themes, such as flora and water imageries, associating the product with environmental friendliness and a healthy lifestyle [58]. The “environmental and social justice” themes, often featuring the company’s commitment to sustainability and eco-friendliness, were also seen, consistent with other tobacco companies’ campaigns using environmental themes to greenwash their products [59], which could be of particular interests to youth, and companies are using greenwash for this purpose [60].

Instagram and Facebook purportedly restrict the marketing content for tobacco products by only allowing companies to post and sell tobacco products to age-restricted audiences [61]. However, social media platforms are generally self-regulated, and age restrictions vary across platforms. It is highly possible for users to bypass age gating and be exposed to age-restricted marketing contents.

To continue marketing a new tobacco product, the company must demonstrate that the marketing would be “appropriate for the protection of the public health” meaning that the benefits to current tobacco product users who might stop using the products outweighs the harms to youth and other nonusers who might start using the products [62]. A significant factor supporting the FDA’s determination that Vuse Solo met the APPH standard was that while current established cigarette users who might use Vuse Solo to switch would prefer the original or tobacco flavor, “existing evidence consistently indicates that use of tobacco-flavored ENDS is less common compared to non-tobacco flavored ENDS among youth.” [63] However, recent studies presented insufficient evidence of any public health benefits associated with Vuse Solo to either adults or youth [64,65]. After data collection and completion of this study, the FDA authorized the marketing of Vuse Alto tobacco-flavored pods and devices [15]. Nevertheless, the findings clearly showed that RJ Reynolds marketed the more popular unauthorized product (Vuse Alto) and that regardless of authorization, products being marketed continue to use advertisements with features that are appealing to youth. Further, given Vuse Alto’s largest market share among all Vuse products and the second largest market share of e-cigarette sales in the United States, and the popularity among high school students [1,66], there is great concern about the marketing of these products and whether the APPH standard is being met.

Our analysis demonstrates that Vuse Alto social media marketing is likely targeting and appealing to adolescents, and therefore likely to promote use by minors. The FDA stated it intends to prioritize enforcement against unauthorized e-cigarettes such as Vuse Alto that are “targeted to minors or whose marketing is likely to promote use” by minors [11]. Based on our findings and the FDA’s stated priorities, the FDA should enforce against the marketing of Vuse Alto e-cigarette products which is appealing to and likely promoting use by minors. In future MGOs, the FDA should explicitly state that the authorized products of the subject MGO must not be comarketed, explicitly or implicitly, with unauthorized products, especially on media channels that would increase exposure to adolescents, including social media.

### Conclusions

Social media posts for unauthorized Vuse products use themes that are appealing to adolescents and are likely to promote youth use, including creativity and innovation, self-expression, arts and music, nature, technology, and alcohol imagery. The findings are important for the authorization of all e-cigarettes. Marketing of brands that are and are not authorized by the FDA is likely indistinguishable to users and potential users, especially youth. As such, it is not APPH to allow comarketing or marketing of brands that are not authorized by the FDA, and it violates the conditions underlying the Vuse Solo MGOs, thus potentially providing the FDA sufficient grounds to issue an order withdrawing the MGOs. The FDA should abide by its own enforcement priorities and enforce against marketing of unauthorized products whose marketing is likely appealing to adolescents and promote youth use. The results of this study suggest that stronger enforcement is generally needed against thousands of other unauthorized products that are currently marketed to youth. Finally, the FDA should prohibit companies from explicitly or implicitly comarketing unauthorized products alongside authorized products.

## Abbreviations

APPH: appropriate for the protection of the public health
CAY: Content Appealing to Youth
e-cigarette: electronic cigarette
FDA: Food and Drug Administration
MGO: marketing granted order
PMTA: premarket tobacco product application
RJRV: RJ Reynolds Vapor Company
